# Exponential advances in Forest Genetics: the past 25 years and the next 25 weeks

**DOI:** 10.1186/1753-6561-5-S7-K1

**Published:** 2011-09-13

**Authors:** Ronald Sederoff

**Affiliations:** 1 Forest Biotechnology Group, North Carolina State University, USA

## 

A history of Forest Biotechnology will be briefly reviewed, from the first cloned tree gene, transgenic trees, DNA markers, genetic maps, QTLs, marker assisted breeding to genome sequencing. Perspectives of newer methods and their applications will be discussed, including integration of genetics and RNA-seq, metabolomics, and proteomics. New applications may be anticipated or expected for breeding, vegetative propagation, phenotypic plasticity, epigenetics, symbiosis, adaptation to biotic and abiotic stress and the directed evolution of some forest tree species.

Forest Biotechnology began over 25 years ago with the advent of new technology of molecular genetics able to advance the fundamental and applied science of forest trees. Methods of gene cloning and gene transfer held the promise of bypassing the long generation times that made genetic analysis and breeding so difficult. This vision has come to pass and the presentations of this meeting show how far we have come. Today, we are deeply involved in the next generation of genetic technology, a second revolution, using genomic sciences, based on the technology and concepts derived from whole genome sequencing, with the same objective, advancing the fundamental and applied science of forest trees.

The path ahead appears to have a third phase, based on an extension of the genomic paradigm, to systems biology and predictive modeling, directed to the same basic goals. Each new phase has been highly integrative, the first bringing molecular genetics into many traditional disciplines. The second phase, integrated areas of molecular and quantitative genetics with high throughput processing and bioinformatics. What appears to be the third phase will extend this paradigm to incorporate the biochemistry of proteomics, the chemistry of metabolomics and the organization, structure and dynamics of cell biology and whole plant physiology. This integrated paradigm further incorporates an engineering perspective and describes the integrated data as predictive mathematical models, subject to experimental test and manipulation. Much of this new biology will be at the interface of biology and systems engineering. Biologists will learn to think like engineers, and engineers will learn a lot of biology. In time, the distinctions between these disciplines may be lost.

What makes a tree a tree? We now know that a relatively small number of genes regulate the woody phenotype, because a small combination of gene mutations can produce a woody form of Arabidopsis. Similarly, small numbers of genes regulate key aspects of tree dormancy. Much remains to be determined regarding the control of tree growth and development, and little is known about the genetic basis of the wide diversity in morphology, metabolism and adaptation. Beyond the descriptive understanding of tree phenotypes and their diversity, lies the evolutionary question, how did the diversity of trees get to be that way.

Comparative genomics has been a powerful tool to derive gene function by comparisons of woody and herbaceous plants, because a large number of functions are conserved. A major barrier to a broad understanding of trees and all other plants still lies in the lack of information on the function of the majority of plant genes. The functions of most genes are known only by superficial annotation based on the time and place of transcript expression, or response to an external stress. Even for many genes of presumed known function, we do not know if that is the only function or the primary function. Even as more genes are subjected to gain of function /loss of function tests, interpretation suffers from effects of redundancy and lethality.

In the near future, genomic scale integration of processes at the levels of molecules, cells, tissues and organisms, as part of a systems biology approach, will bring about progress toward this goal. This goal appears daunting. One challenge comes from the knowledge of epistasis, or genetic interactions, that indicate substantial complexity even for simple traits. QTL analysis provides a general picture of this complexity, with a frequent genetic architecture of a small number of genes of large effects with a larger number of genes with small effects. Thanks to genomics and computers, the quantitative effects of a large number of genes may now be studied at the molecular level as is being done for genomic selection.

Traditionally, new concepts and technology developed in model systems see earlier applications in medicine and agriculture than in forestry. The lag time may be decreasing. The paradigm for genomics was established by the human genome project, where the draft sequence was initiated in 1989 and a first draft released in 2000. The sequence of Arabidopsis was completed in 2000, rice in 2002, and the first tree genome, black cottonwood, completed in 2006. We see application of new technology to traditional problems, in some cases with great success, and in others with great promise.

Only a few years after gene transfer was demonstrated in model plants, an herbicide resistance gene was inserted into poplar and a tree regenerated. Deployment of genetically modified crops has met with resistance, however, today worldwide; there are about 120 million hectares of GM crops, with half in the USA. GM trees are still highly regulated in the US. In China, GM trees have been widely deployed in the last decade. GM trees still face substantial resistance in the US and more so in Europe.

Chestnut serves as an important model for a Genetically Modified Tree. The prospects of development of a blight resistant American chestnut appear quite good based on a combination of backcross breeding; map based cloning and genetic transformation. A genome sequence for Chinese chestnut is in progress (Carlson et al.). The use of gene transfer for chestnut restoration is compelling because of the high value of the species, and because GM may be required to restore the species. The restoration of the American chestnut would have great value to Appalachian forest ecosystems, as a resource of high value wood, and potentially for bioenergy. The restoration would serve as an example for the application of biotechnology and genomics to many valuable species currently threatened by pests and pathogens or climate change.

